# Multi-Target Tracking Based on a Combined Attention Mechanism and Occlusion Sensing in a Behavior-Analysis System

**DOI:** 10.3390/s23062956

**Published:** 2023-03-08

**Authors:** Xiaolong Zhou, Sixian Chan, Chenhao Qiu, Xiaodan Jiang, Tinglong Tang

**Affiliations:** 1 College of Electrical and Information Engineering at Quzhou University, Quzhou 324000, China; 2 Key Lab of Spatial Data Mining & Information Sharing of Ministry of Education, Fuzhou 350108, China; 3 College of Computer Science and Technology, Zhejiang University of Technology, Hangzhou 310023, China; 4 Hubei Key Laboratory of Intelligent Vision-Based Monitoring for Hydroelectric Engineering, The College of Computer and Information at China Three Gorges University, Yichang 443002, China

**Keywords:** multi-object tracking, object detection, data association, object occlusion, attention mechanism

## Abstract

Multi-object tracking (MOT) is a topic of great interest in the field of computer vision, which is essential in smart behavior-analysis systems for healthcare, such as human-flow monitoring, crime analysis, and behavior warnings. Most MOT methods achieve stability by combining object-detection and re-identification networks. However, MOT requires high efficiency and accuracy in complex environments with occlusions and interference. This often increases the algorithm’s complexity, affects the speed of tracking calculations, and reduces real-time performance. In this paper, we present an improved MOT method combining an attention mechanism and occlusion sensing as a solution. A convolutional block attention module (CBAM) calculates the weights of space and channel attention from the feature map. The attention weights are used to fuse the feature maps to extract adaptively robust object representations. An occlusion-sensing module detects an object’s occlusion, and the appearance characteristics of an occluded object are not updated. This can enhance the model’s ability to extract object features and improve appearance feature pollution caused by the short-term occlusion of an object. Experiments on public datasets demonstrate the competitive performance of the proposed method compared with the state-of-the-art MOT methods. The experimental results show that our method has powerful data association capability, e.g., 73.2% MOTA and 73.9% IDF1 on the MOT17 dataset.

## 1. Introduction

Artificial intelligence is widely used in the field of healthcare [1,2]. Specifically, researchers focus on human behavior analysis based on multi-target tracking (MOT) for healthcare systems [3]. MOT is a topic of interest in the field of computer vision, which has broad prospects in fields, including intelligent video monitoring [4,5,6], assisted driving [7,8,9], smart agriculture [10,11], and behavior analysis [12,13,14]. The main task is to track multiple objects in a video sequence, assign unique identifiers (IDs) to each object, maintain the stability of identity when occlusion and interaction occur, and finally obtain the object’s motion track.

The main problems to be solved include an object’s occlusion, interference of similar objects, and mutual influence between multiple objects. As the tracking environment is complex and changeable, and the characteristics of tracked objects are similar, the performance of MOT systems is limited by their ability to distinguish the appearance characteristics of multiple objects and keep them stable [15].

A detection-based MOT algorithm structure can be divided into detectors, trackers, and classifications; such algorithms can be either detection-based (DBT) or detector-free (DFT), according to whether an image must be detected. The present study focuses on improving (1) the speed of extracting different object features as well as the stability of distinguishing between object features; and (2) the accuracy of associations between classifiers under different association methods to balance the tracking accuracy and operational efficiency [16].

MOT algorithms can be online or offline, depending on whether a video sequence is progressively tracked frame by frame, i.e., whether real-time video streams can be analyzed and predicted. Among online algorithms, Bewley proposed SORT [17] to divide a tracking algorithm into object-detection modules, using Faster R-CNN [18] to complete object detection for input video, and Kalman filters and Hungarian algorithms to determine if an object in different frames is the same. To address the instability of the SORT algorithm in the association module, DeepSORT [19] introduced appearance model-assisted data association, which improved the accuracy of the data association and reduced the probability of a failed association due to occlusion and other problems.

Zhang et al. [20] proposed an improved DeepSORT algorithm based on YOLOv5 for MOT, improving the efficiency of tracking. Considering that the detection model and embedding model exist in the DeepSORT-like algorithm and that the two models run independently, reducing the efficiency of MOT, Wang et al. [21] proposed JDE, which integrated the detection and embedding models for joint embedding learning, which was more efficient than a separate detection and embedding Model (SDE), such as DeepSORT. The image resolution and running speed reached 1088 × 688 and 18.8 FPS, respectively, which was close to the requirement of real-time speed (20 FPS). Yoo et al. [22] designed an object constraint learning method to raise the tracking efficiency. Boragule et al. [23] advanced a pixel-guided method to combine the joint-detection and tracking task for MOT.

There are two difficulties in the current research of MOT algorithms:

(1) In complex environments, such as environments with many occlusions, the algorithm’s tracking needs to be improved for the same object.
(2) Operational efficiency. The application scenario of multi-target tracking can determine that it needs to run at a speed close to or even beyond the real-time speed (20 FPS). Specifically for online algorithms, high running-speed requirements are proposed.

This paper presents an improved tracking method based on joint detection and embedding learning (JDE), and makes the following contributions: (1) To unify spatial and channel features, a CBAM extracts the attention weights of space and channel dimensions from the feature map. An adaptive fusion of feature maps using attention weights enhances the model’s ability to extract object features. (2) To solve the problem of temporary occlusion of the detected object, an occlusion-detection module adaptively determines the current object occlusion situation. If this stops the updating of the obscured object’s appearance features, the resultant contamination is improved. (3) The proposed method achieved excellent tracking performance on public datasets.

The rest of this paper is organized as follows. Section 2 describes related work. Section 3 introduces the proposed method in detail. Some experimental results are discussed in Section 4 followed by our concluding remarks in Section 5.

## 2. Related Work

### 2.1. MOT Algorithm

There are two- and one-step MOT algorithms, depending on whether a single network is used to predict the detection information and re-identification (Re-ID) of an object.

#### 2.1.1. Two-Step MOT Algorithm

The two-step algorithm takes the output of the object-detection task as the input of the object re-identification task. Hence, two tasks are processed separately using different network models, which requires serial execution.

For example, based on SORT [17], DeepSORT [19] introduces an appearance model based on a CNN [18], which adds the ability to extract the appearance-feature information of the object in the input image, and enhances the robustness of the algorithm during object tracking. The integration of appearance-feature information improves the model’s handling of long-term occlusion, which reduces the error object ID switching that occurs during tracking. The Tracktor++V2 [24] utilizes the bounding box to predict the location of the target in the next frame, thus, converting the detector into one tracker. However, due to the use of two networks to obtain object-detection information and object-appearance characteristics, DeepSort is more efficient than SORT, and serially scheduling two tasks limits efficiency.

#### 2.1.2. One-Step MOT Algorithm

Two-step MOT algorithms use two networks to obtain object-detection information and object-appearance characteristics. The JDE [21] algorithm is proposed to integrate the object-detection model and the appearance-embedding model into the same network. The FairMOT [25] applies the CenterNet [26] to create two homogeneous branches of detection and embedding for predicting pixel-level objectness information. The TransCenter [27] is implemented the multi-scale and pixel-level query and object-centered heatmap via linking them frame by frame based on the transformer network.

This allows a single network to complete the extraction of these two types of information in forward propagation to improve the algorithm’s efficiency. The performance improvement of one-stage and two-stage algorithms mainly manifests in four aspects: speed, accuracy, model complexity, and data augmentation. Compared to two-stage algorithms, one-stage algorithms are faster since they directly perform dense bounding-box prediction on the entire image, avoiding the calculation of both object-detection and object-tracking steps, thus, being suitable for applications with high real-time requirements.

However, one-stage algorithms usually have lower accuracy compared with two-stage algorithms, as they need to perform dense bounding-box prediction on the entire image and are prone to false positives and false negatives. Additionally, one-stage algorithms usually have simpler network structures and fewer parameters than two-stage algorithms, while the latter needs to design both object detection and object tracking networks, thus, being relatively more complex. Finally, one-stage algorithms usually adopt some data augmentation techniques, such as data augmentation and adaptive sampling, to improve the algorithm generalization and robustness, further enhancing the algorithm performance.

### 2.2. Attention Mechanism

Attention is an inherent mechanism of human vision. When the human eye looks at an object or scene, the distribution of attention is different according to the object or scene. Such a mechanism can help humans quickly obtain critical information from the environment and allows for careful observation of detailed information about objects. The attention mechanism in in-depth learning learns from the human attention mechanism and is widely used in in-depth learning tasks, such as image classification and natural language processing. Depending on the scope, attention mechanisms can be categorized as channel attention [28], spatial attention [29], or mixed attention [30].

Spatial attention mechanisms arise because, for an input image, part of the area is unrelated to the identification or segmentation task, and so only the area related to the task must be processed. It can compute the spatial information of the input image while preserving the key information and suppressing non-key information. A representative model of spatial attention mechanisms is the spatial transformer network (STN) [29], proposed by Google DeepMind, which learns from the input to select a preprocessing operation suitable to a task.

For visual tasks, the input image has both spatial and channel dimensions, and the ability of the network to extract the feature information from the image can be effectively improved by studying the dependency between channels in the feature map. SENet [28] is a representative channel attention mechanism model, which compresses the spatial dimension of the input signature graph, preserves the channel dimension, generates weights for each channel through the network, learns to adjust them for each channel during training, and multiplies the generated weight matrix by the original input signature graph, which enlarges the signature information of important channels, suppresses the signature information of less-important channels, and improves the efficiency of the network at extracting signature information.

The mixed-attention mechanism combines spatial and channel information in a hybrid attention mechanism, ignoring the intrinsic relationship between features and failing to consider both spatial and channel characteristics. For example, the CBAM [30] and dual-attention network [31] are representative models.

### 2.3. Shortcomings or Research Gaps

Based on the current related work, we summarize the current shortcomings of multi-object tracking as follows:

1. Robustness: Multi-object tracking algorithms still need to improve their robustness to external factors, such as lighting changes, occlusions, and motion blur.
2. Long-term tracking: Long-term tracking involves cross-frame object re-identification and model updates, and there are still some challenges, such as model drift, occlusions, and motion blur.
3. Object re-identification: Object re-identification is one of the key technologies of multi-object tracking; however, there are still certain issues, such as object deformations and viewpoint changes.
4. Algorithm efficiency: Multi-object tracking algorithms usually need to process a large amount of data, and fast and efficient algorithms are needed for real-time applications.

## 3. Proposed Method

### 3.1. FairMOT Framework

Researchers have found that one-step models, such as JDE [21] use an anchor-based detection network [32] that results in inconsistencies between the appearance embedding features extracted from the anchor point and the real object during training, thereby, resulting in a decrease in the tracking accuracy. Therefore, Zhang et al. [25] proposed a one-step MOT algorithm, FairMOT, based on an anchorless frame.

#### 3.1.1. DLAseg-Based Backbone

Deep layer aggregation (DLA) [33] can iteratively aggregate specific information about a network structure with higher accuracy as the number of parameters decreases, similar to a pyramid network. The DLAseg [26] network used by the FairMOT algorithm introduces more hop connections based on the DLA network, enabling more information to be shared between lower and higher features. To alleviate the problem of detecting critical points and aligning objects, deformable convolution improves the information extraction capability of upsampling operations, thus, enabling the network to dynamically adjust the sensing field to the size of the object.

**Figure 1:**
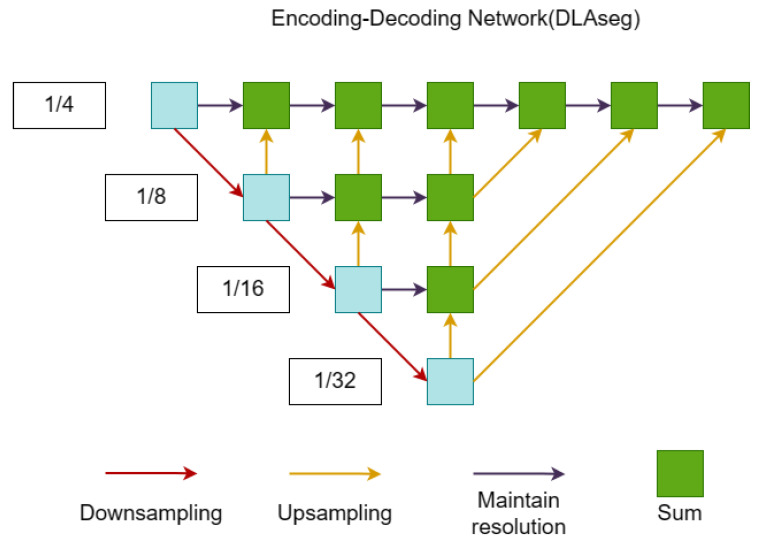
DLAseg network structure of the algorithm backbone.

Different from traditional convolutional kernels, the shape and position of deformable convolutional kernels are learned, making them better suited to irregular shapes and position variations of the targets. In addition, deformable convolutions can reduce the number of parameters, enhance the generalization ability of the model, and improve its performance. The structure of the DLAseg network, as shown in Figure 1, is based on DLA34 and introduces a variant network after deformable convolution.

In the input and output part of the network, we express the size of the input image as $H_{image}\times W_{image}$, and the output signature graph has the dimensions C×H×W, where $H=H_{image}/4$ and $W=W_{image}/4$.

#### 3.1.2. Object Detection Branch

**Figure 2:**
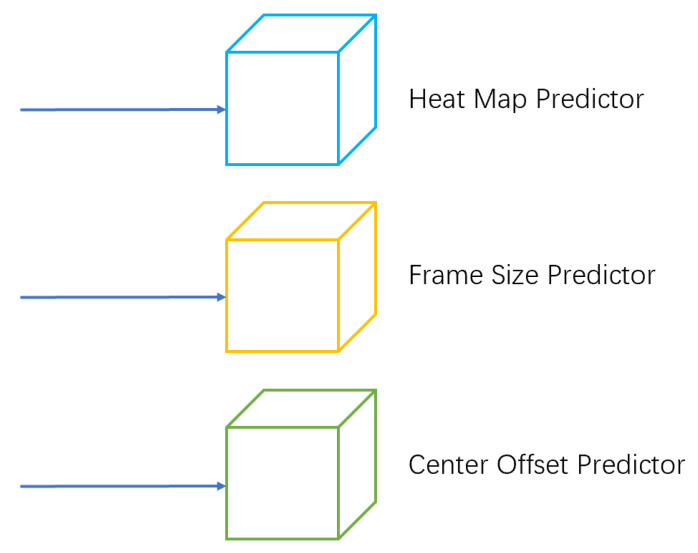
The schematic diagram of the object-detection branch, which consists of three predictors: the heat map predictor, the bounding box predictor, and the center offset predictor.

The FairMOT algorithm adds three parallel predictors to the object detection part of the tracking task—a thermogram predictor, frame size predictor, and center offset predictor, as shown in Figure 2—based on the DLASEG network, each consisting of a convolution of 256 channels with a convolution core sizes of 3×3 and 1×1.

The thermogram predictor predicts the center position of the object. The output characteristic diagram of the thermogram is H×W×1. If the thermogram collapses with the center of the real object, the response of the location in the output characteristic diagram is 1. The box size predictor predicts the size of the object detection bounding box at each object location.

The center offset predictor locates the object more accurately in the image. As the resolution of the feature map is one-fourth that of the original image, a step size of 4 in the feature map introduces an error of up to 4 pixels in the original image. With the introduction of a center offset predictor, the offset of each pixel point in the image relative to the true object center point can be estimated from the output feature map of the predictor, thereby, mitigating the effects of the error on the sampling.

#### 3.1.3. Object Recognition Branch

**Figure 3:**
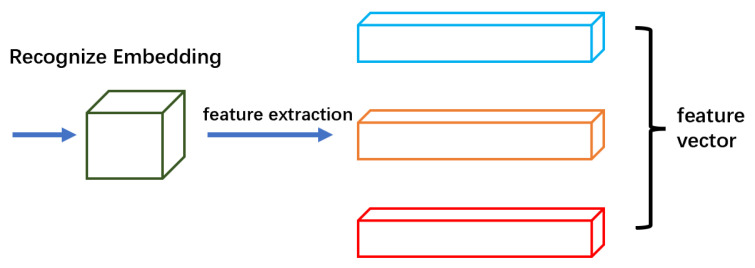
Object recognition branch: we apply a convolution layer with 128 kernels on top of the backbone feature to extract re-ID features for each location.

The object recognition branch, as shown in Figure 3, generates visual features that distinguish different tracking objects in a single task. In an ideal preset, the degree of similarity between different objects output by the object recognition branch should be less than that of the same object.

### 3.2. Convolutional Attention Module

**Figure 4:**
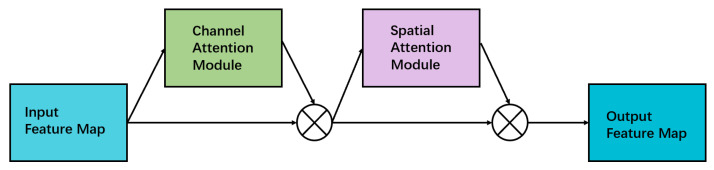
The attention-mechanism structure of convolution blocks.

The CBAM [30], as shown in Figure 4, is a lightweight attention module. Based on the SE module of SENet [28], the attention module of the second dimension, i.e., the spatial dimension, is added. Hence, the CBAM can be divided into channel attention and spatial attention modules.

The CBAM extracts features from multiple channels or spaces by blending them with convolution. From the perspective of the change of the signature graph, the calculation can be expressed as

(1) $$F^{\prime }=M_{c}\left(F\right)⊗F,$$

(2) $$F^{\prime \prime }=M_{s}\left(F^{\prime }\right)⊗F^{\prime },$$

where ***F*** represents the input characteristic map, $M_{c}$ is the calculation of channel attention; $M_{s}$ is the calculation based on spatial attention; and $F^{\prime }$ and $F^{\prime \prime }$ are the output characteristic maps after calculating the channel and spatial attention, respectively.

**Figure 5:**
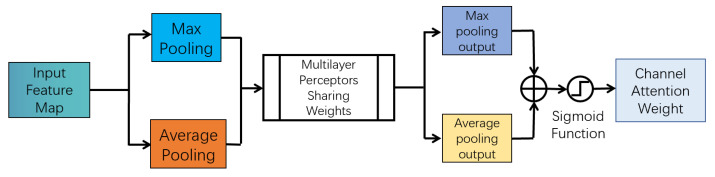
Structure of the channel attention module.

The channel attention module, as shown in Figure 5, performs maximum and average pooling on the input signature map, and uses a shared weighted multilayer perception machine to learn and predict. After adding the two output matrices, channel attention weights are obtained through the sigmoid activation function [34].

The above operations of the channel attention module can be expressed as Equation (3).

(3) $$M_{c}\left(F\right)=\sigma \left(W_{1}\left(W_{0}\left(F_{\mathrm{avg}\;}^{c}\right)\right)+W_{1}\left(W_{0}\left(F_{\max}^{c}\right)\right)\right),$$

where σ represents the Sigmoid activation function, $W_{0/1}$ represents two convolution operations in a multilayer perception machine, $F_{\mathrm{avg}}^{c}$ represents the average pooling characteristics for channel attention, and $F_{max}^{c}$ represents the maximum pooling characteristics for channel attention.

**Figure 6:**
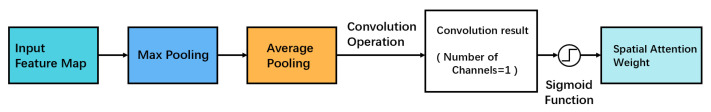
Structure of the spatial attention module.

The spatial attention module, as shown in Figure 6, maximizes the pooling operation on the input feature map and then averages the pooling operation. The experiments [30] indicate that performing only a single pooling operation results in significant information loss. Using the parallel connection of average and max pooling reduces the amount of lost information compared to single pooling, thus, resulting in better performance. The result is convolved with a convolution core of 7×7 and obtains the spatial attention weight after using the sigmoid activation function on the result.

**Figure 7:**
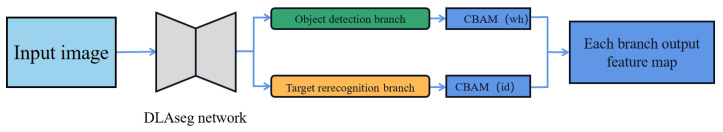
Network structure diagram after CBAM joining.

The operations of the spatial attention module can be expressed as Equation (4).

(4) $$M_{s}\left(F\right)=\sigma \left(\int^{7\times 7}\left(F_{\mathrm{avg}\;}^{s};F_{\max\;}^{s}\right)\right),$$

where σ represents the sigmoid activation function, $\int^{7\times 7}$ represents convolution with a convolution core of 7×7, $F_{avg}^{s}$ represents the average pooling feature for spatial attention, and $F_{max}^{s}$ represents the maximum pooling feature for spatial attention. The CBAM is mainly added in the DLAseg [26] backbone network and prediction branch; the location affects its effects. Experiments show that adding the CBAM after forecasting branches can best improve the comprehensive performance of multiple indicators. The experimental process and results can be seen in Section 4. The network structure after adding the CBAM is shown in Figure 7.

In the prediction branch, the number of channels of the heatmap predictor, center offset predictor, frame size, and object recognition predictor are 1, 2, 4, and 128, respectively. In the CBAM, the effect of applying attention to the feature map of low channel numbers is not good. Therefore, the CBAM is inserted in the frame size predictor and object recognition predictor in this paper.

### 3.3. Occlusion-Sensing Module

**Figure 8:**
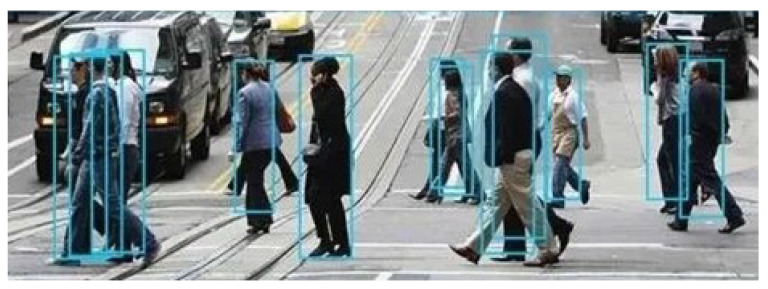
Pedestrian occlusion problem in pedestrian tracking.

**Figure 9:**
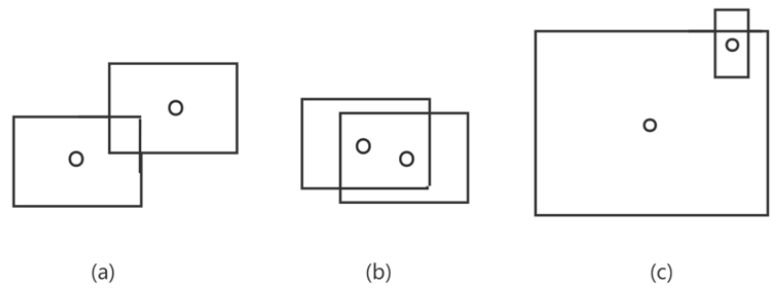
Pedestrian occlusion diagram under different conditions. (**a**) Partial occlusion of two pedestrians with similar size. (**b**) Large occlusion of two pedestrians with similar size. (**c**) Occlusion of two pedestrians with large different sizes.

For occlusion detection (as shown in Figure 8), the traditional IoU crossover ratio algorithm [31] calculates the coincidence ratio as the ratio of the intersection area of the two detection boxes to that of their union, and filters the objects that meet the requirements by setting a threshold value. This works well when the proportions of the sizes between the objects are similar. However, in pedestrian-tracking tasks, the sizes of pedestrian-detection boxes may vary greatly depending on the distance from the camera, which greatly reduces the effect of the IoU algorithm. In Figure 9a,b, the ratios computed by IoU better reflect the overlap of the two objects, whose frames are of similar size. In case (c), however, the size difference between the object boxes is large, and the IoU calculation does not accurately show the occlusion of small objects.

Based on the traditional IoU crossover ratio algorithm [31], we introduce the judgment of the object’s center point. When judging two object frames for occlusion, if the center point of one object frame is within the coordinate range of another frame, the object is determined to be occluded. Assuming two object-detection boxes, $b_{1}$ and $b_{2}$, with center points $c_{1}$ and $c_{2}$, respectively, IoU indicates the result of the cross-ratio calculation of the two object-detection boxes, and F indicates whether there is occlusion, where 1 indicates occlusion, and 0 indicates none, i.e.,

(5) $$F=\left\{\begin{array}{cc} 0, & \;\mathrm{IoU}\;>=0\;\mathrm{and}\;c_{1}\notin b_{2}\;\mathrm{and}\;c_{2}\notin b_{1}\\ 1, & \;\mathrm{else}\; \end{array}\right..$$

**Figure 10:**
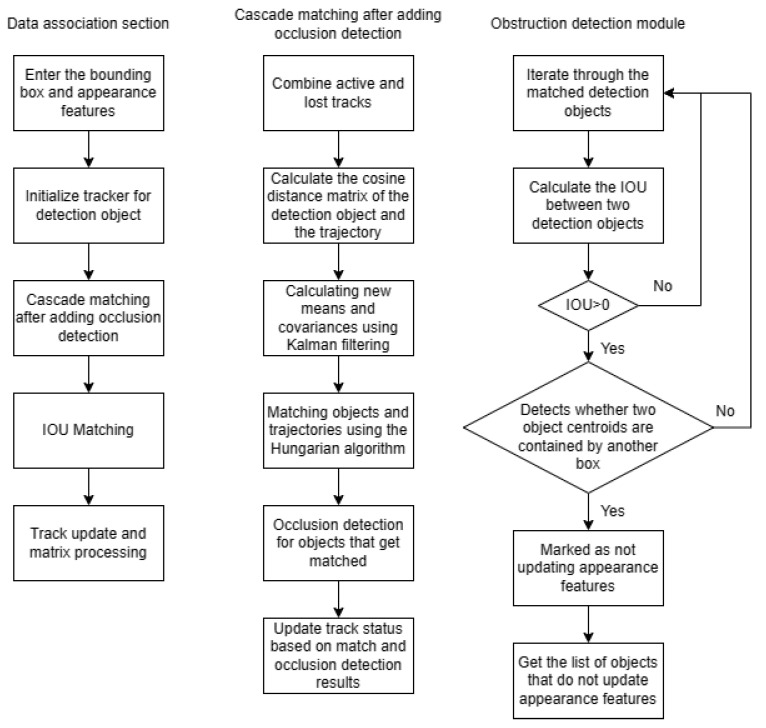
Schematic diagram of the data-association part after adding the occlusion-detection module.

In FairMOT [25], the detected object is matched with the track reserved in the tracker by cascade matching and IoU matching in the association part. The most successful association of the object occurs in the cascade matching part; thus, adding the occlusion-detection module here can obtain a good result. The improved tracker flow is shown in Figure 10, where the bold module represents modification after adding the occlusion-detection module.

### 3.4. Loss Function

#### 3.4.1. Heatmap Loss Function

The size of the heatmap is *H* × *W* × 1. If it collapses with the center of a real object, the response at that location is 1. The response decays exponentially with the distance between the location in the heatmap and the object center.

For each GT box $b^{i}=\left(x_{1}^{i},y_{1}^{i},x_{2}^{i},y_{2}^{i}\right)$, the object center is $\left(c_{x}^{i},c_{y}^{i}\right)$, where $c_{x}^{i}=\left(x_{1}^{i}+x_{2}^{i}\right)/2$ and $c_{y}^{i}=\left(y_{1}^{i}+y_{2}^{i}\right)/2$. Then, the coordinates of the point on the feature map are divided by the step size, i.e., $\left(\tilde{c_{x}^{l}},\tilde{c_{y}^{l}}\right)=\left(\left\lfloor \frac{c_{x}^{i}}{4}\right\rfloor ,\left\lfloor \frac{c_{y}^{i}}{4}\right\rfloor \right)$. The heatmap response calculation for location (x,y) is

(6) $$M_{xy}=\sum_{i=1}^{N}\exp^{-\frac{{\left(x-\tilde{c_{x}^{l}}\right)}^{2}+{\left(y-\tilde{c_{y}^{l}}\right)}^{2}}{2\sigma_{c}^{2}}},$$

where *N* is the number of objects in the image, and $\sigma_{c}$ is the standard deviation.

The loss function is defined as a pixel-level logistic regression with focal loss,

(7) $$\begin{array}{ccc} & & {\hat{N}}_{xy}=1-{\hat{M}}_{xy},\\ & & L_{heatmap}=-\frac{1}{N}\sum_{xy}\left\{\begin{array}{c} {\hat{N}}_{xy}^{\alpha }\log{\hat{M}}_{xy},\;\mathrm{if}\;M_{xy}=1\\ {\hat{N}}_{xy}^{\beta }{\hat{M}}_{xy}^{\alpha }\log{\hat{N}}_{xy},\mathrm{otherwise}, \end{array}\right. \end{array}$$

where $\hat{M}$ is the heatmap for prediction, and α and β are preset loss parameters.

#### 3.4.2. Box Offset and Size Prediction Loss Function

The frame offset predictor is used to locate the object more accurately in the image. The predictor estimates the constant offset of each pixel point from the object center to mitigate the effect of downsampling. The size predictor estimates the size of the object bounding box at each location. As the step size of the signature map is 4, a nonnegligible error of up to 4 pixels is introduced.

The outputs of the box offset predictor and size predictor are $\hat{O}\in R^{2\times H\times W}$ and $\hat{S}\in R^{2\times H\times W}$, respectively. The size of each GT box $b^{i}=\left(x_{1}^{i},y_{1}^{i},x_{2}^{i},y_{2}^{i}\right)$, can be calculated as $s^{i}=\left(x_{2}^{i}-x_{1}^{i},y_{2}^{i}-y_{1}^{i}\right)$, and the offset can be similarly calculated as $o^{i}=\left(\left(c_{x}^{i}\right)/4,\left(c_{y}^{i}\right)/4\right)-\left(\left[\left(c_{x}^{i}\right)/4\right],\left[\left(c_{y}^{i}\right)/4\right]\right)$. The offset and size of the corresponding location are expressed as $\hat{O}$ and $\hat{S}$, respectively, and the $l_{1}$ loss is added to the two predictors,

(8) $$L_{box}=\sum_{i=1}^{N}{∥o^{i}-{\hat{o}}^{i}∥}_{1}+\lambda_{s}{∥s^{i}-{\hat{s}}^{i}∥}_{1},$$

where $\lambda_{s}$ is a weight factor, which is set to 0.1 in the original CenterNet [26] network.

#### 3.4.3. Object Recognition Loss Function

The resulting feature graph is $E\in R^{128\times H\times W}$, and the object recognition feature is extracted from the object whose center is located at (x,y) is $E_{x,y}\in R^{128}$. The object center $\left(c_{x}^{i},c_{y}^{i}\right)$ is obtained for each GT box $b^{i}=\left(x_{1}^{i},y_{1}^{i},x_{2}^{i},y_{2}^{i}\right)$ in the image. An eigenvector $E_{x^{i},y^{i}}$ can be extracted and mapped to a class distribution vector P=p(k),k∈[1,K] using a fully connected layer and a softmax operation. The one-hot of the GT class label is represented as $L^{i}\left(k\right)$, and the object recognition loss is

(9) $$L_{i\;\mathrm{dentity}\;}=-\sum_{i=1}^{N}\sum_{k=1}^{K}L^{i}\left(k\right)\log\left(p\left(k\right)\right),$$

where *K* is the number of categories.

#### 3.4.4. Overall Loss Function

The loss of the detection and recognition branches is added to the total loss, and two tasks of indeterminate loss [35], automatic balance detection and recognition, are added to calculate the total loss, as shown in Equations (10) and (11).

(10) $$L_{detection}=L_{heatmap}+L_{box},$$

(11) $$L_{\mathrm{total}\;}=\frac{1}{2}\left(\frac{1}{e^{w_{1}}}L_{\mathrm{detection}\;}+\frac{1}{e^{w_{2}}}L_{i\;\mathrm{dentity}\;}+w_{1}+w_{2}\right),$$

where $w_{1}$ and $w_{2}$ are learnable parameters used to balance the detection and recognition tasks.

## 4. Experiments and Analysis

### 4.1. Experimental Datasets

#### 4.1.1. MOT Series Dataset

The MOT Series dataset is an open dataset proposed by the MOTChallenge, focusing on pedestrian-tracking tasks. The picture files of the training and test sets are exposed, the labels of the training set are exposed, and the labels of the test set are retained. The series is divided into the MOT15 [36], MOT16 [37], MOT17, and MOT20 [38] datasets. MOT15 is modified from other old datasets. MOT16 and MOT17 are new datasets, in which pedestrians are much more crowded. MOT20 is the largest and most dense dataset in the series. We used MOT Series datasets for validation and testing.

#### 4.1.2. CrowdHuman Dataset

The CrowdHuman dataset [39] is an open dataset that is publicly available and focuses on pedestrian detection tasks. The dataset contains 15,000 pictures of the training set, each with header, body, and visible bounding boxes labeled on each pedestrian object. The test dataset does not expose a label file. We used the CrowdHuman dataset for training.

#### 4.1.3. MIX Dataset

MIX is a hybrid dataset based on datasets proposed by the author of the JDE algorithm. It includes six datasets: Caltech Pedestrian, CityPersons, CUHK-SYSU, PRW, ETHZ, and MOT17. MIX is mainly used for training MOT task models. We used these datasets for training.

### 4.2. Evaluation Metrics

We thoroughly benchmarked our method using five standard evaluation metrics, the main ones being MOTA [40] and IDF1 [41]. MOTA measures the overall performance of the tracker by evaluating mistakes from three sources—namely, mostly tracked object (MT), mostly lost object (ML), and identity switching (IDs).The IDF1 is concerned with the quality of assigning identities with unity in the detection quality of the identity.

### 4.3. Analysis of Experimental Results

Based on the FairMOT [25] research and improvements, we used the improved algorithm system to train on the MOT dataset, used the final model to test on the MOT series dataset, and analyzed the results.

#### 4.3.1. Training Process and Results

We conducted experiments that were trained using PyTorch on a server with two NVIDIA RTX 2080Ti GPUs, an 8-core Intel Xeon Silver 4110 CPU and 32 GB memory. Based on the pretraining model, the improved algorithm trained 60 batches using the CrowdHuman dataset, trained 30 batches on the MIX dataset using the trained model, and tested the experimental results using the resulting final model. The hyperparameters were set as follows: the batch size was set to 6 for CrowdHuman training and 12 for MIX training.

**Figure 11:**
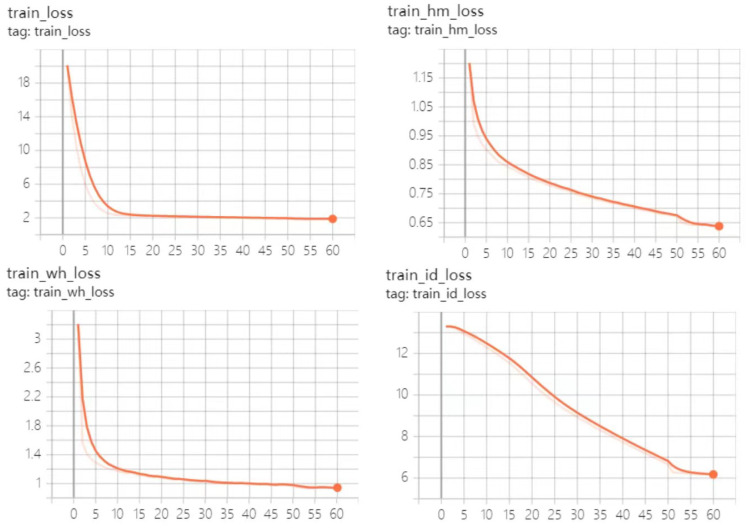
Training results for the model trained for 60 epochs on the CrowdHuman dataset.

**Figure 12:**
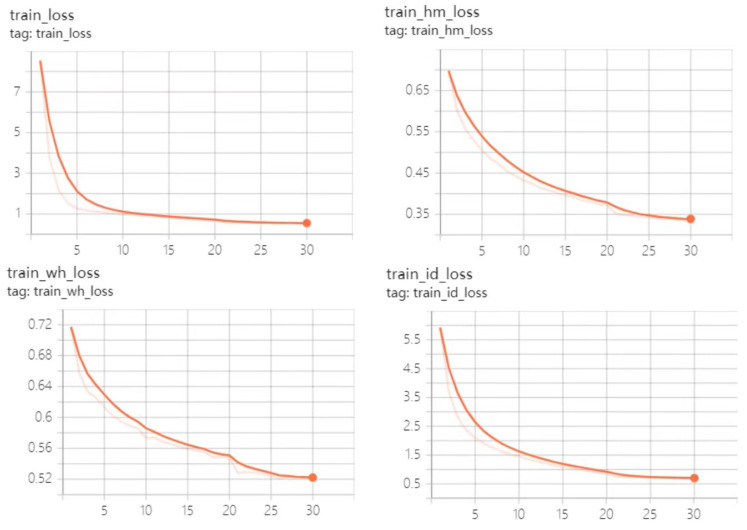
Training results for the model trained for 30 epochs on the MIX dataset.

The initial learning rate was set to 0.0001. The dataset load thread (num workers) was set to 8. The relationships between loss function curves and training batches are shown in Figure 11 and Figure 12. The changes of each loss function during 60 batches of training using the CrowdHuman dataset based on the pretraining model are shown in Figure 11. The changes of each loss function during 30 batches of training batches, using the MIX dataset to the model trained on the CrowdHuman dataset, are illustrated in Figure 12.

From the results shown in Figure 11 and Figure 12, we can see that at the end of the training, all the loss values of the network were in a convergent state, and the convergence effect was ideal.

#### 4.3.2. Model Comparison Experiment

Table 1 shows the experimental results on the MOT20 dataset using the FairMOT model and the improved model in this paper.

It can be seen in Table 1 that the MOTA [40] and IDF1 [40] indices of the modified model on the MT20 dataset were increased by 1.7% and 1.54%, respectively. Therefore, after the introduction of the CBAM and the pedestrian occlusion-detection module, the model improved the ability to extract object-detection information and appearance features, produced more accurate feature information, and improved the tracking accuracy of the model.

In terms of the number of MT and ML indicators, for the same object in the tracking process, the stability of the tracker was improved due to the improvement of the feature information. At the same time, the short-term pedestrian occlusion-detection module reduced the problem of appearance feature pollution by stopping appearance feature updates after occlusion detection. It also improved the robustness of tracking.

Table 2 shows the experimental results on MOT16. Most algorithms do not publish the specific number of *MT* and *ML* indicators but instead publish the percentage of indicators. *MT* and *ML* from the experimental results of this algorithm were converted to percentages in Table 2 and subsequent comparisons.

From Table 2, we can see that the proposed model was improved on the MOTA16 dataset, especially MOTA and IDF1, which are the most critical measures of MOT, by 0.1% and 1.3%, respectively, indicating enhanced tracking robustness. Significantly, the number of ID switches in this model is significantly lower than that in FairMOT, indicating an improved predictive effect of the tracker after adding an attention mechanism to the object recognition branch and the pedestrian occlusion-detection module. The lower number of IDs makes the model tracking result more useful in practical applications. The improved model in this paper has excellent levels of all indicators when compared with the other models in the table. The results on the test setting of the MOT17 are shown in Table 3.

### 4.4. Ablation Experiments

**Figure 13:**
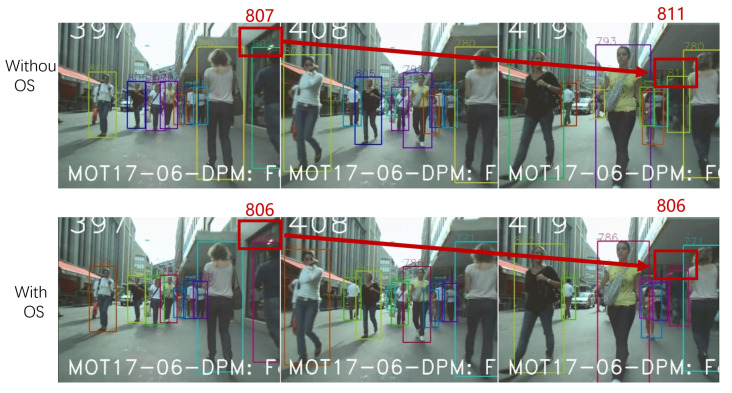
Visualization of the tracking results: without OS vs. with OS.

To analyze the effectiveness of the different components of our proposed framework, we also designed a series of baseline methods for comparison. The MIX dataset was set as the training dataset, and the MOT20 training set was applied as the test dataset. The ablation study of occlusion sensing is reported in Table 4. OS indicates the occlusion-sensing module. Figure 13 shows the visualization of the tracking results on MOT17. The results without OS lose the blocked object (807ID changes to 811ID as a new object), while the OS can deal with this situation. Both of them illustrate the effectiveness of the occlusion-sensing module.

## 5. Conclusions

In this paper, we proposed a novel approach for multi-target tracking that utilizes a combined attention mechanism and occlusion sensing. The motivation behind our approach was to tackle the challenges posed by object occlusions, which can significantly affect the accuracy and robustness of object-tracking systems.

To this end, we designed a convolution block attention module that calculates the weights of space and channel attention from the feature map. The attention weights were then used to fuse the feature maps, which allowed for the adaptive extraction of robust object representations. Additionally, we introduced an occlusion-sensing module that is capable of detecting occlusions. Once an occlusion occurs, the appearance of the occluded object was not updated to ensure the purity of the object. To evaluate the effectiveness of our method, we conducted experiments on three widely used datasets: MOT16, MOT17, and MOT20.

The experimental results show that our method achieved different degrees of improvement on these datasets, demonstrating its accuracy and robustness in multi-target tracking scenarios. Specifically, our approach outperformed state-of-the-art methods in terms of multiple evaluation metrics, such as MOTA and IDF1. These results validate the effectiveness of our approach in handling challenging scenarios, such as occlusions, and improve the overall performance of multi-target tracking systems. 

## Figures and Tables

**Table 1:** Test results of the improved algorithm on the test set of the MOT20 dataset.

	MOTA ↑	IDF1 ↑	MT ↑	ML ↓	IDs ↓
FairMOT [25]	61.00%	65.3%	66.80%	7.6%	5243
Ours	**62.70**%	**66.84**%	**66.92** %	7.63%	**4806**

**Table 2:** Experimental results of the improved algorithm on the MOT16 dataset.

	MOTA ↑	IDF1 ↑	MT ↑	ML ↓	IDs ↓
EAMTT [42]	52.5%	53.3%	19.9%	34.9%	910
SORT [17]	59.8%	53.8%	25.4%	22.7%	1423
VMaxx [43]	62.6%	49.2%	32.7%	21.1%	1389
TubeTK [44]	64.0%	59.4%	33.5%	19.4%	1117
JDE [21]	64.4%	55.8%	35.4%	20.0%	1544
CNNMTT [35]	65.2%	62.2%	32.4%	21.3%	946
CTrackV1 [45]	67.6%	57.2%	32.9%	23.1%	5529
FairMOT [25]	74.7%	73.4%	**44.7**%	**15.9**%	1074
Ours	**74.8**%	**74.7**%	41.5%	19.0%	**819**

**Table 3:** Experimental results of the improved algorithm on the MOT17 dataset.

	MOTA ↑	IDF1 ↑	MT ↑	ML ↓	IDs ↓
SST [46]	52.40%	49.50%	21.40%	30.70%	8431
TubeTK [44]	63.00%	58.60%	31.20%	19.90%	4137
CTrackV1 [45]	66.60%	57.40%	32.20%	24.20%	5529
CenterTrack [47]	67.80%	64.70%	34.60%	24.60%	2583
FairMOT [25]	73.10%	72.70%	**41.10%**	**19.00%**	2964
Ours	**73.20%**	**73.90%**	39.70%	21.00%	**2553**

**Table 4:** Experimental ablation study of occlusion sensing on the MOT20 dataset.

	MOTA ↑	IDF1 ↑	MT ↑	ML ↓	IDs ↓
Without OS	61.21%	64.70%	66.63%	8.60%	5568
With OS	**62.70**%	**66.84**%	**66.92**%	**7.63**%	**4806**

## Data Availability

This study did not report any data. We used public data for research.
